# Detection of mobile colistin resistance genes *mcr-9.1* and *mcr-10.1* in *Enterobacter asburiae* from Ecuadorian children

**DOI:** 10.1128/mra.00313-24

**Published:** 2024-08-20

**Authors:** Sara G. Cifuentes, Jay Graham, Gabriel Trueba, Paúl A. Cádenas

**Affiliations:** 1 Instituto de Microbiología, Colegio de Ciencias Biológicas y Ambientales, Universidad San Francisco de Quito USFQ, Quito, Pichincha, Ecuador; 2 Berkeley School of Public Health, University of California, Berkeley, California, USA; Montana State University, Bozeman, Montana, USA

**Keywords:** enterobacter, *mcr*, colistin

## Abstract

Colistin is one of the last-line treatments for multi-drug resistant Gram-negative bacterial infections. The emergence of mobile colistin resistance genes has driven global concern and triggered the need for surveillance. Our report reveals the identification of *mcr-9.1* and *mcr-10.1* in Ecuador by employing a proximity ligation technique.

## ANNOUNCEMENT

The emergence of mobile colistin resistance genes endangers the treatment of infections caused by multi-drug resistant Gram-negative bacteria (1, 2). The mobile colistin resistance gene *mcr*-1 was initially discovered in China in 2015 (3), followed by detection in Ecuador in 2016 (4); however, no additional variants of the *mcr* gene have been reported in the country since then (5–11). During a metagenomics study (12), *mcr-9.1* and *mcr-10.1* genes were identified. This study received approval from the Ethics Committee for Research in Human Beings at Universidad San Francisco de Quito USFQ (IRB# 2017-178M) and the Office for Protection of Human Subjects at the University of California, Berkeley (IRB# 2019-02-11803).

Two fecal samples were collected from two healthy young boys, aged 1 and 5, as part of a repeated measures study that recruited 600 children from 2018 to 2021 (13). The collection methods and transport conditions for samples have been detailed in a previous study (12). We employed a culture-independent approach, dividing the specimens into DNA extraction and crosslinking aliquots. Genomic DNA was isolated employing the Qiagen QIAamp Fast DNA Stool Mini Kit (cat. no.51604, Qiagen) following a modified protocol (14) and stored at −80°C until further analyses. The second aliquot was crosslinked with 1% formaldehyde for 20 minutes (15). DNA and crosslinked samples were sent to Phase Genomics for ProxiMeta full-service analysis. The complete protocol has been detailed previously (16), and default parameters were applied unless otherwise specified. Briefly, the shotgun library was prepared using the Watchmaker DNA Library Prep Kit (cat. no. 7K0103-096, Watchmaker Genomics, USA), and the proximity ligation library was created using the ProxiMeta Hi-C kit (cat. no. KT5045, Phase Genomics, USA). Shotgun metagenomic and Hi-C libraries were sequenced on an Illumina NovaSeqX platform (2 × 150 bp paired-end reads), yielding 708,033,774 and 293,351,862 read pairs for the two shotgun metagenomic and Hi-C libraries, respectively. Fastp v0.20.1 was used to preprocess and control the FASTQ data quality (17). Shotgun metagenomic assemblies were generated and assessed using Megahit v1.2.9 (18) and MataQUAST v5.2.0 (19), respectively. Hi-C reads were mapped to the metagenomic assemblies using BWA-MEM (0.7.17-r1198-dirty) (20, 21). Instead of using a conventional metagenomic binning approach, contigs were clustered into genome clusters with the ProxiMeta platform (Phase Genomics, USA) (22). Mash was used to compare genome clusters with NCBI RefSeq genomes, and CheckM (23) evaluated the quality of the genomes in terms of completeness and contamination. Antimicrobial resistance genes (ARGs) were detected using AMRFinderPlus (https://www.ncbi.nlm.nih.gov/pathogens/antimicrobial-resistance/AMRFinder/) and ResFinder v4.0 (24) (https://cge.food.dtu.dk/services/ResFinder-4.1/). Both colistin resistance genes showed 100% identity and 100% template coverage. Metagenome deconvolution analysis identified these ARGs on plasmid contigs, although we could not characterize the plasmids harboring these ARGs using PlasmidFinder v.2.1 (https://cge.food.dtu.dk/services/PlasmidFinder/) with the available contigs. Table 1 presents an overview of the genome assembly statistics, colistin resistance, co-resistances, and the bacterial host. This report highlights the importance and the feasibility of community-level metagenomic surveillance to detect the spread of mobile resistance genes that pose a risk to public health.

**TABLE 1:** Summary of data for two metagenome-assembled genomes of *Enterobacter asburiae* strains carrying mobile colistin resistance and other mobile and genomic ARGs

Bin ID/sample name	Taxonomy	Genome size	Genome completion (%)	Contig N50	Number of contigs	GC (%)	Mobile *mcr* gene	Other mobile antimicrobial resistance genes	Genomic antimicrobial resistance genes	SRA accession no./assembly accession no.
bin_3/HC45	*Enterobacter asburiae L1*	4,081,523	91.26	19,822	273	56.13	*mcr-10.1*	*qnrB19* *tet(A*)	*blaACT-4* *blaACT-7* *oqxA oqxB*	SRS20620200 JBEOKR000000000.1
bin_4/HC32	*Enterobacter asburiae B*	4,677,763	94.85	20,261	145	55.65	*mcr-9.1*	N/A	*blaACT-6* *fosA oqxA oqxB*	SRS20620198 JBEOKS000000000.1

## Data Availability

This Whole Genome Shotgun project has been deposited at DDBJ/ENA/GenBank as BioProject PRJNA1082298 (see Table 1 for more accession numbers).
